# The impact of pressure and temperature on the quality of suspended red blood cells: An ex vivo simulation study

**DOI:** 10.1111/tme.13141

**Published:** 2025-05-02

**Authors:** Chunyu Feng, Rui Fan, Haimei Ma, Huan Zhang

**Affiliations:** ^1^ School of Clinical Medicine Tsinghua University Beijing China; ^2^ Department of Anesthesiology Beijing Tsinghua Changgung Hospital Beijing China; ^3^ Department of Blood Transfusion Beijing Tsinghua Changgung Hospital Beijing China

**Keywords:** blood transfusion, fluid warming, haemolysis, heating

## Abstract

**Objective:**

This study aims to explore the impact of pressurisation and simultaneous warming at a combination of 50 kPa and 46°C on the quality of suspended red blood cells in an ex vivo environment.

**Background:**

During massive rapid blood transfusion, pressure and temperature‐controlled blood warming devices are often used to prevent hypothermia caused by the infusion of large amounts of cold blood. If the pressure and temperature are not properly applied during this process, it can endanger the patient's life safety.

**Methods/Materials:**

400 mL of human suspended red blood cells stored at 2–6°C were subjected to pressure and simultaneous warming at a combination of 50 kPa and 46°C. Changes in blood temperature and blood quality‐related indicators before and after warming under pressure were detected, with the procedure repeated six times.

**Results:**

In the ex vivo simulated test environment, there were no statistically significant differences in routine blood indicators, biochemical indicators, and hemolysis rates of suspended red blood cells before and after pressure and warming transfusion at 50 kPa pressure and 46°C temperature (P>0.05). There were no significant changes in osmotic fragility after pressure and warming transfusion, and no obvious hemolysis was observed in the morphology of suspended red blood cells under an electron microscope.

**Conclusion:**

In the ex vivo simulated test environment, pressure and warming transfusion at 50 kPa pressure and 46°C temperature had no significant impact on blood quality, and the blood quality met the standards for the use of blood products.

## 1 INTRODUCTION

Haemorrhage in war, disaster, trauma, and surgery is a major cause of patient mortality. Rapid and massive blood transfusion is an important measure to replenish effective circulating blood volume and save patients' lives.^1^, ^2^, ^3^ At the same time, since whole blood and red blood cells are stored at 2–6°C,^4^ rapid transfusion must be accompanied by physiologically appropriate warming measures to avoid hypothermia caused by the rapid infusion of cold blood, which can affect metabolism, leading to coagulation abnormalities, tissue metabolism disorders, malignant arrhythmias, electrolyte disturbances, and other severe complications.^5^, ^6^, ^7^

Studies^8^ have shown that when red blood cells are pressurised to 80 kPa during simple pressure infusion, no significant haemolysis occurs. It is also generally believed that blood should be warmed to the human body temperature of 37°C,^9^, ^10^, ^11^, ^12^, ^13^ and studies have indicated^11^, ^14^, ^15^, ^16^ that warming blood to 45–46°C also almost does not lead to red blood cell haemolysis.

However, there are few studies that have tested the impact of simultaneous pressure and warming during blood transfusion on the quality of suspended red blood cells, and no studies have set the blood warmer temperature above 46°C during pressure and warming blood transfusion. Therefore, this study intends to use the most commonly used pressure bag in clinical practice for pressurisation, with the maximum pressure of 50 kPa that the bag can withstand, and set the blood warmer temperature to 46°C. The study will simulate the warming and pressurisation of suspended red blood cells ex vivo, and detect changes in the quality of suspended red blood cells before and after warming and pressurisation. This will provide guidance for clinical warming and pressurisation blood transfusion and offer data support for the further development of warming and pressurisation blood transfusion equipment.

## 2 METHODS

### 2.1 Instrumentation

Blood transfusion pressure bag (Smith, Model: MX4705); Disposable transfusion set (Mindray, Shenzhen, China); Fully automatic haematology analyser (Mindray, Model: BC‐5390); Fully automatic biochemical analyser (Roche, C8000); Medical centrifuge (Baiyang, Model: BY‐320C); Multifunctional microplate reader (PerkinElmer, Model: PE EnVision); Scanning electron microscope (FEI, Model: Quanta 200); Transmission electron microscope (Hitachi, Model: H‐7650); Pressure and temperature control equipment (provided by the Laboratory of Precision Instrumentation, Department of Precision Instruments, Tsinghua University) including: air pump, pneumatic pressure regulator, pneumatic solenoid valve, air pressure sensor, flow sensor, platinum resistance, thermocouple temperature sensor, constant temperature bath, spiral copper tube, IPT measurement and control system, IPTMonitor software, Navicat software.

### 2.2 The preparation method for suspended red blood cells

Whole blood is mixed evenly with CPDA‐1 preservative solution, then centrifuged to prepare suspended red blood cells, without undergoing leukoreduction. The storage time for suspended red blood cells before heating is 7–10 days.

### 2.3 Consumables

Disposable blood transfusion set; Intravenous catheter (14G); Free haemoglobin detection kit (o‐toluidine colorimetric method) (Reagen Bio, Batch number: 0816A23); NaCl particles (Aobo Star, Batch number: 20230208); Disposable vacuum blood collection tubes: routine blood tube 2 mL (Kangjian, Batch number: 0077230401, anticoagulant: EDTA‐K2); Disposable vacuum blood collection tubes: additive‐free tube 2 mL (Kangjian, Batch number: 0012221201); Physiological saline.

### 2.4 Experimental steps

Simulate the pressure and temperature‐controlled blood transfusion process, collect blood samples, and record the blood flow rate and temperature changes before and after pressure and temperature‐controlled transfusion. Take 400 mL of human suspended red blood cells stored at 4°C, simulate the actual clinical scenario, let it sit at room temperature for 10 min, gently mix, and use the pressure and temperature‐controlled device to perform combined pressure and warming treatment at 50 kPa pressure and 46°C temperature. Before the pressure and temperature‐controlled transfusion, 6 mL of blood sample was injected into two routine blood tubes (2 mL each) and one additive‐free tube (2 mL) as pre‐transfusion samples. The temperature was subsequently measured and mixed for testing. During transfusion, the real‐time blood flow rate (data collected every 0.5 s) was measured until the blood flow rate stabilised. Subsequently, a post‐transfusion 6 mL of blood sample was collected and injected into two routine blood tubes (2 mL each) and one additive‐free tube (2 mL), with temperature measurement and mixing for testing, as post‐transfusion samples. The post‐transfusion samples were compared to the pre‐transfusion samples, the latter which acted as a control. This process was repeated six times with different donors to reduce the impact of individual differences on the experimental results.

### 2.5 Determination of routine blood parameters

After shaking the routine blood tubes well, use a fully automatic haematology analyser to detect red blood cell count (RBC), haemoglobin content (HGB), haematocrit (HCT), mean corpuscular haemoglobin (MCH), and mean corpuscular haemoglobin concentration (MCHC).

### 2.6 Determination of biochemical parameters

After centrifuging the additive‐free tube with a medical centrifuge (1610× g) for 5 min, collect the upper plasma and use a fully automatic biochemical analyser to detect the levels of potassium (K+), indirect bilirubin (IBIL), and lactate dehydrogenase (LDH).

### 2.7 Determination of free haemoglobin and calculation of haemolysis rate

The detection method for free haemoglobin (FHb) follows the instructions of the reagent kit, and the hemolysis rate (P) is calculated using the formula,^17^ P = [(1 − HCT) × CFHb/CHb] × 100%, where HCT is the haematocrit, CFHb is the concentration of free haemoglobin in the plasma or supernatant (g/L), and CHb is the haemoglobin concentration (g/L).

### 2.8 Determination of red blood cell osmotic fragility

Weigh 1 g of dry NaCl and dissolve it in 100 mL of distilled water, prepare nine groups of 1 mL NaCl solutions with different concentrations ranging from 0.28% to 0.60% with a gradient of 0.04%, and use 1 mL 0.90% physiological saline as a control. Add 30 μL of EDTA‐K2 anticoagulated whole blood to each tube, gently mix, let it sit for 2 h, and then observe the haemolysis of the upper liquid.

### 2.9 Preparation and observation of scanning electron microscope samples

Cells for scanning electron microscopy (SEM) were fixed in a mixture of paraformaldehyde (2%) and glutaraldehyde (2.5%), then washed 4 times with PB buffer (0.1 M), followed by ethanol dehydration in graded solutions (50%, 70%, 80%, 90%, 100%, 100%, 100%) for 10 min each and taken to critical point. The cells were studied under the environmental scanning electron microscope (ESEM) Quanta 200 (FEI, Hillsboro, America).

### 2.10 Preparation and observation of transmission electron microscope samples

Cells initially fixed in a mixture of paraformaldehyde (2%) and glutaraldehyde (2.5%) for 1 h and then washed 4 times with PB buffer (0.1 M). Cells underwent post‐fixation with Osmium tetroxide (1%) and Tetrapotassium hexacyanoferrate trihydrate (1.5%) for 1 h at 23°C, followed by ethanol dehydration in graded solutions (50%, 70%, 80%, 90%, 100%, 100%, 100%, 100%) for 10 min each. Then, 1,2‐Epoxypropane twice for 10 min each and gradient infiltration with a mixture of 1,2‐Epoxypropane and Epon 812 resin for 8 h (SPI, America). Subsequently, Pure Epon 812 twice and polymerising in an oven (60°C). Blocks of polymerised resin were sectioned using a Leica EM UC7 ultramicrotome (Wetzlar, Germany). Ultra‐thin sections (80 nm) were mounted and dried on coated copper grids. Sections were stained on‐grid with 2% uranyl acetate (20 min) and lead citrate (5 min). Imaging was carried out using an HT 7800 transmission electron microscope (Hitachi, Tokyo, Japan).

### 2.11 Statistical methods

Data were analysed using the SPSS 25.0 statistical software package. A two‐tailed *p*‐value of less than 0.05 was considered statistically significant for all tests. Continuous variables were tested for normality. Normally distributed variables are expressed as the mean ± standard deviation, and intergroup comparisons were made using the paired‐samples t‐test. Non‐normally distributed variables are expressed as the median M (P25, P75), and intergroup comparisons were made using the Wilcoxon signed‐rank test. A *p*‐value of less than 0.05 was considered to indicate a significant difference with statistical significance.

## 3 RESULTS

### 3.1 Changes in blood temperature before and after pressure and warming transfusion

After pressure and temperature‐controlled transfusion, the blood flow rate can reach a rate of 291 mL/min. When transfused at a rate of 291 mL/min, the blood temperature can be increased on average from 9.73 to 24.88°C.

### 3.2 Changes in routine blood parameters before and after pressure and temperature‐controlled transfusion

There was no statistically significant change in the routine blood parameters of red blood cells before and after pressure and temperature‐controlled transfusion (*p* > 0.05), as shown in Table 1. There were no obvious changes in the routine blood parameters before and after pressure and temperature‐controlled transfusion.

**TABLE 1:** Changes in routine blood parameters before and after pressure and temperature controlled transfusion.

	Control group	Pressure and temperature‐controlled group	*z*‐value/*t*‐value	*p*‐value
RBC (10^12^/L)	6.60 (6.07, 7.42)	6.52 (6.51, 7.48)	0.241	0.809
HGB (g/L)	194.33 ± 2.52	192.00 ± 3.00	1.185	0.289
HCT (%)	60.73 ± 3.40	60.83 ± 1.97	−0.277	0.793
MCH (pg/Cell)	27.10 ± 1.51	26.87 ± 1.69	0.595	0.578
MCHC (g/L)	320.33 ± 6.43	316.00 ± 6.56	0.945	0.388

### 3.3 Changes in blood biochemical parameters before and after pressure and temperature‐controlled transfusion

The differences in changes of blood biochemical parameters before and after pressure and temperature‐controlled transfusion were not statistically significant (*p* > 0.05), as shown in Table 2.

**TABLE 2:** Changes in biochemical parameters before and after pressure and temperature‐controlled transfusion.

	Control group	Pressure and temperature‐controlled group	*z*‐value/*t*‐value	*p*‐value
K+ (mmol/L)	45.74 ± 3.20	45.65 ± 3.33	0.002	0.999
LDH (U/L)	485.00 ± 347.46	590.67 ± 121.71	−1.506	0.192
IBIL (μmol/L)	0.70 (0.38’1.13)	0.95 (0.55, 1.90)	−0.944	0.345
P (%)	0.09 ± 0.00	0.17 ± 0.04	−3.480	0.074
Abnormal cells (%)	8.17 ± 0.68	8.58 ± 0.58	0.955	0.383

### 3.4 Changes in hemolysis rate before and after pressure and temperature–controlled transfusion

The FHb content in the plasma was measured before and after pressure and temperature‐controlled transfusion, and the hemolysis rate was calculated. There was no statistically significant difference in the hemolysis rate before and after pressure and temperature‐controlled transfusion (*p* > 0.05), as shown in Table 2.

### 3.5 Observation of cell morphology under scanning electron microscope

**FIGURE 1:**
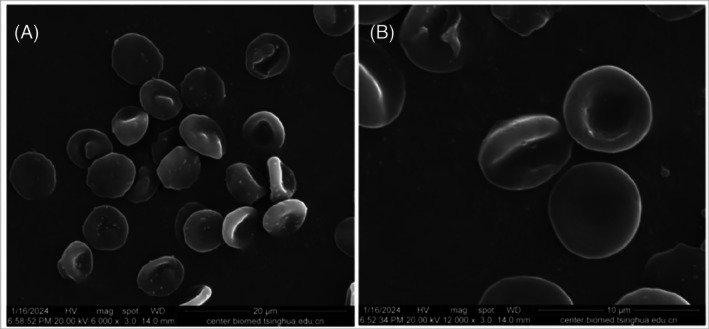
Scanning electron microscopy results of red blood cells without pressure and temperature control. (A) (×6000, Bar = 20 μm), (B) (×12 000, Bar = 10 μm).

**FIGURE 2:**
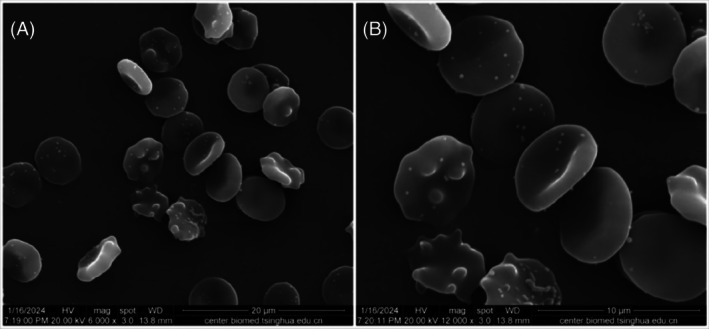
Scanning electron microscopy results of red blood cells after pressure and temperature‐controlled transfusion. (A) (×6000, Bar = 20 μm), (B) (×12 000, Bar = 10 μm).

Under the scanning electron microscope, red blood cells appeared as biconcave discs with smooth surfaces when not subjected to pressure and temperature (Figure 1). After pressure and temperature‐controlled transfusion, cell shrinkage was observed, with irregular edges and an increase in the number of abnormal red blood cells, including ellipsoidal, fusiform, spiculated, and teardrop shapes. Microvesicles were visible on the surface. Figure 2 shows the observation results of red blood cells under the scanning electron microscope after pressure and temperature‐controlled transfusion. A field of view with evenly distributed red blood cells was randomly selected, and more than 300 red blood cells were counted. The percentage of abnormal red blood cells was less than 10%. There was no statistically significant difference before and after pressure and temperature‐controlled transfusion (*p* > 0.05), as shown in Table 2.

### 3.6 Observation of cell morphology changes under transmission electron microscopy

**FIGURE 3:**
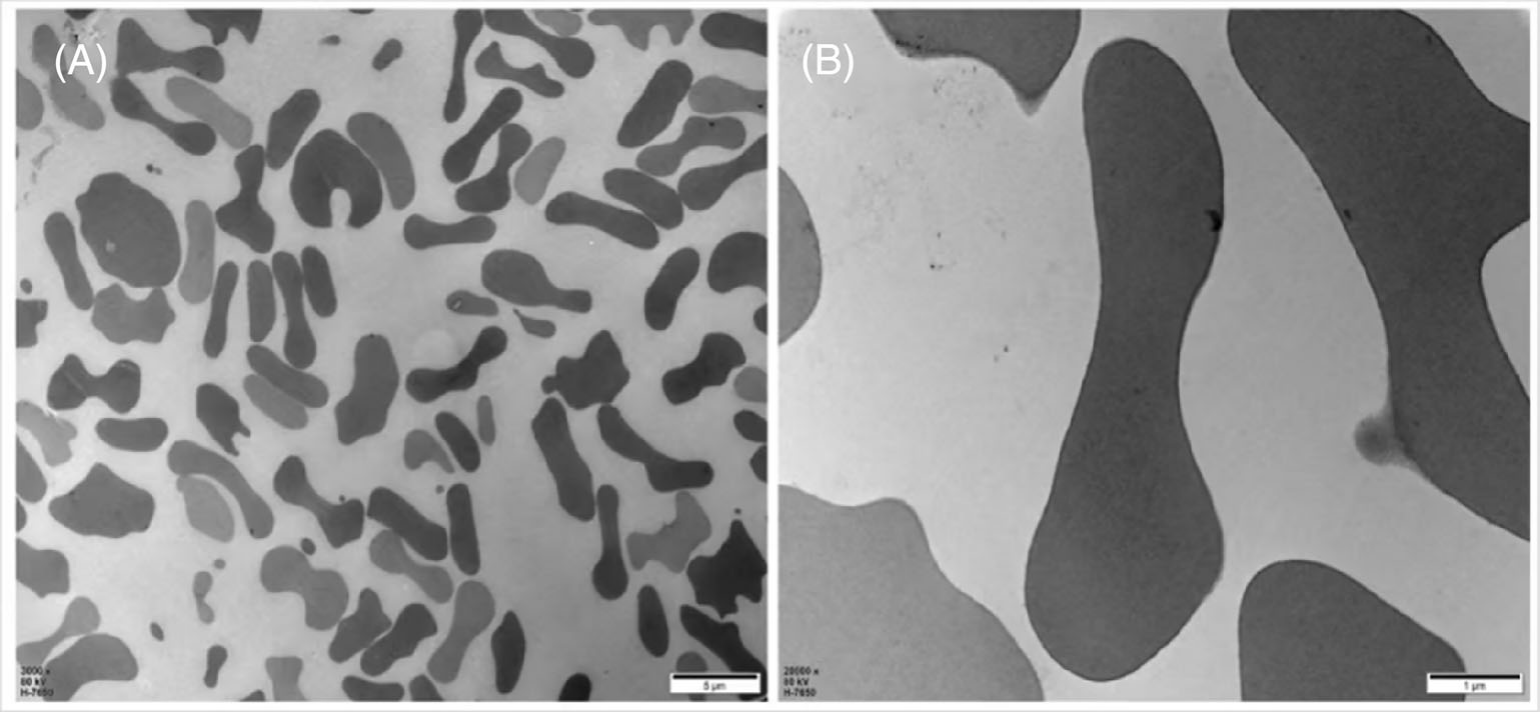
Transmission electron microscopy results of red blood cells without pressure and temperature control. (A) (×3000, Bar = 5 μm), (B) (×20 000, Bar = 1 μm).

**FIGURE 4:**
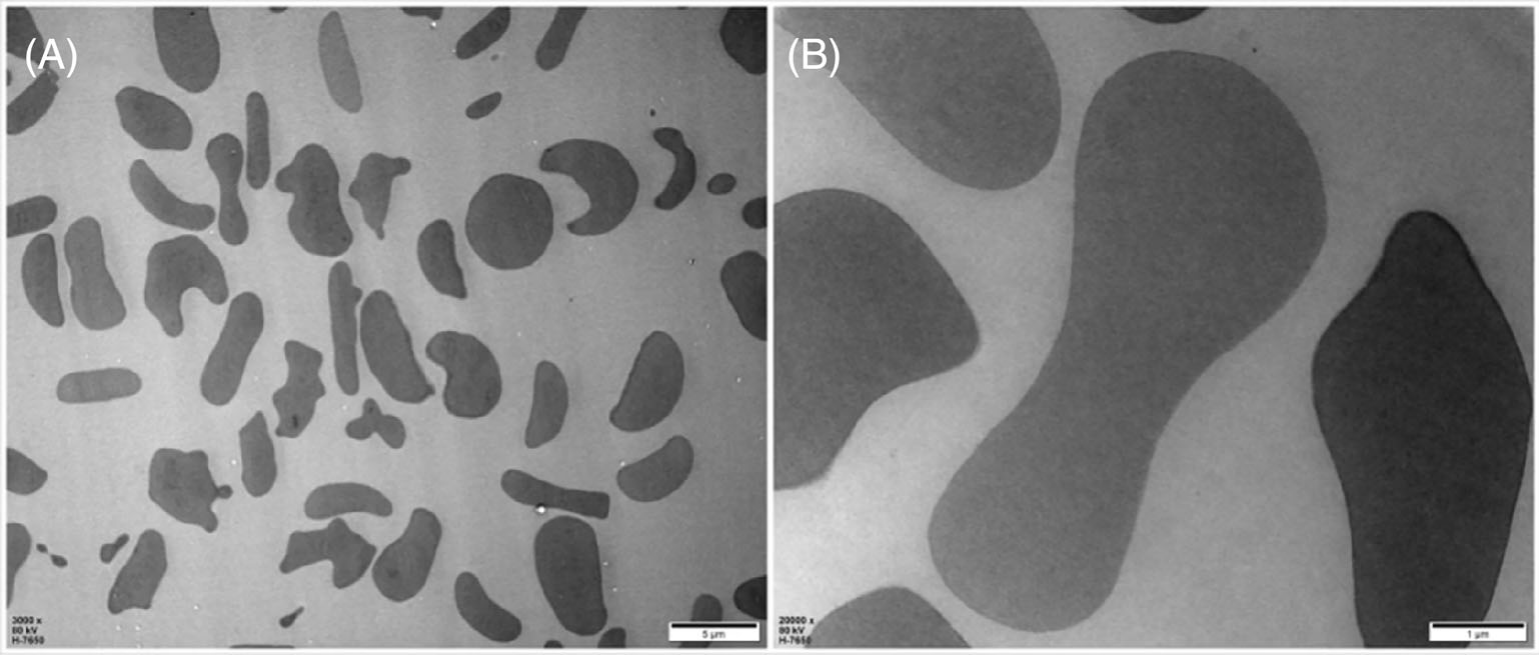
Transmission electron microscopy results of red blood cells after pressure and temperature‐controlled transfusion. (A) (×3000, Bar = 5 μm), (B) (×20 000, Bar = 1 μm).

Normal red blood cells exhibit different shapes due to different cross‐sections, mostly typical biconcave disc shapes, but also including round, dumbbell‐shaped, and irregular forms. The cell membrane is dense, with a clear cytoskeletal structure and uniformly consistent mesh, as seen in Figure 3. Figure 4 shows the results observed under transmission electron microscopy after pressure and temperature‐controlled transfusion, with a higher number of atypical red blood cells, uneven electron density, and disruption of the reticular structure with uneven mesh.

### 3.7 Changes in red blood cell osmotic fragility before and after pressure and temperature‐controlled transfusion

Red blood cell osmotic fragility was tested before and after pressure and temperature‐controlled transfusion. After a 2‐h standstill, hemolysis was observed at a NaCl concentration of 0.52%, and complete hemolysis occurred at a concentration of 0.44%. There was not much difference before and after pressure and temperature‐controlled transfusion. After pressure and temperature‐controlled transfusion, the red blood cells' resistance to hypotonic solutions decreased, and osmotic fragility slightly increased, but there was no significant red blood cell hemolysis.

**FIGURE 5:**
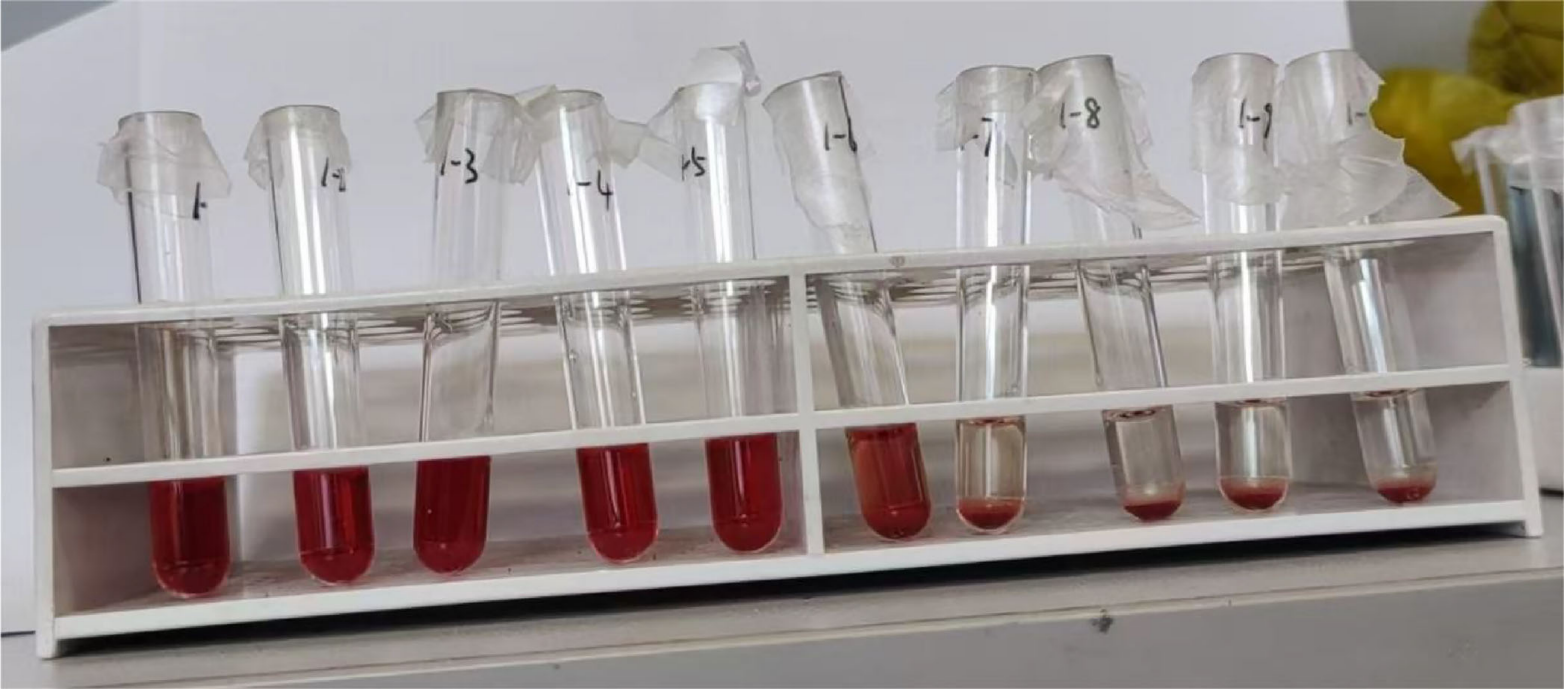
Non‐warmed and non‐pressurised group.

**FIGURE 6:**
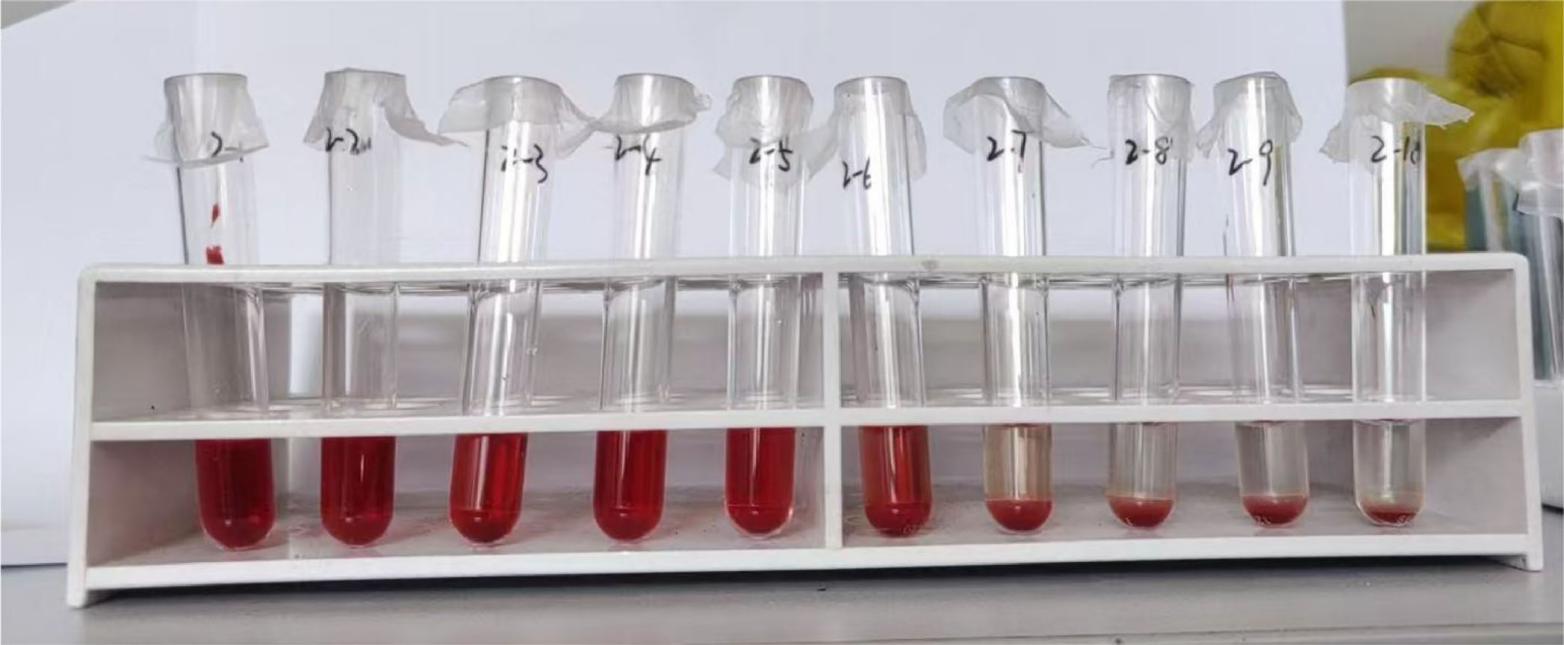
Warmed and pressurised group.

Tubes 1–9 represent 9 different concentrations of 1 mL NaCl solution with a gradient of 0.04% ranging from 0.28% to 0.60%. Tube 10 contains 1 mL of 0.90% saline. Figure 5 shows the results for the non‐warmed and non‐pressurised group, while Figure 6 shows the results for the warmed and pressurised group. The osmotic fragility did not change significantly before and after the pressure and temperature‐controlled infusion.

## 4 DISCUSSION

In scenarios such as war, disaster, and surgery, when patients experience massive blood loss, they often exhibit the “lethal triad of trauma,” which includes hypothermia, acidosis, and trauma‐induced coagulopathy (TIC).^18^ At such times, rapid and massive transfusion of fresh blood is crucial for saving patients' lives. However, since blood is often stored in cold environments at 4°C, simple rapid transfusion often leads to a decrease in body temperature, making it particularly important to warm the blood during rapid transfusion.^19^ This process is not absolutely safe; if red blood cells are subjected to improper pressure or temperature during transfusion, it may lead to hemolysis, producing a large number of red blood cell fragments, potassium ions, and other harmful substances, posing a serious threat to patients.^20^, ^21^, ^22^ Studies have shown^11^, ^14^, ^15^, ^16^ that warming blood to 45–46°C almost does not cause red blood cell hemolysis, but it still affects the stability of red blood cells. If a pressure bag is used simultaneously to apply excessive pressure during combined transfusion, it may lead to significant hemolysis of red blood cells. This study found that when the temperature was set to 46°C and the pressure to 50 kPa for pressure and temperature‐controlled transfusion of suspended red blood cells, the temperature of the suspended red blood cells could be increased from an average of 9.73 to 24.88°C, and the impact on red blood cells was minimal in a short time. Electron microscopy observations after pressure and temperature‐controlled transfusion showed an increase in abnormal red blood cells, with destruction of the cell membrane reticular structure and uneven mesh, confirming the deformation and destruction of red blood cells. However, the changes in hemolysis rate and other related indicators before and after pressure and temperature‐controlled transfusion were not statistically significant, thus no meaningful cell hemolysis occurred. This result provides guidance for clinical pressure and temperature‐controlled transfusion and also provides important data support for the further development of pressure and temperature‐controlled equipment by engineers.

In previous studies, Kim^23^ and others set the warming temperature to 39°C and the pressure to 40 kPa for pressure and temperature‐controlled transfusion of red blood cells, and detected the percentage of hemolysis before and after the transfusion. The results showed no significant increase in the percentage of hemolysis before and after the transfusion. By detecting the temperature of the red blood cells at the outlet, when the red blood cell unit was first warmed to 23.2°C at room temperature before pressure and temperature‐controlled transfusion, the outlet temperature could reach 38.1°C after the transfusion. Mateer^8^ and others found that when the blood warmer was set to 36.7°C and pressure and temperature‐controlled transfusion was performed at pressures of 40 and 80 kPa, there was a slight increase (about 10%) in free haemoglobin before and after the transfusion, but it was not statistically significant. When the temperature of the red blood cell unit at the inlet before pressure and temperature‐controlled transfusion was 13°C, the outlet temperatures after the transfusion were 27.5 and 25.3°C, respectively. Thomas G. Poder^24^ and others mainly observed the percentage of hemolysis as the main indicator. The study showed that there was no statistically significant difference between blood heated at 41.5°C with 20 kPa pressure and blood with 40 kPa pressure. In this study, before pressure and temperature‐controlled transfusion, the cold red blood cell units (i.e., 4°C) were first warmed at room temperature (i.e., 23–24°C) to 16.7°C. After being set to a blood warmer at 41.5°C, the red blood cell temperatures reached 37.1 and 33.65°C, respectively.

Compared with previous studies on pressure and temperature‐controlled transfusion, this study used a heating temperature above 43°C. Considering that the highest warming temperature that most blood warmers currently sold by manufacturers can reach does not exceed 43°C, this study is particularly important for the further development of pressure and temperature‐controlled equipment. In addition, the blood used in this study was within the clinical use period, and the indicators related to red blood cells were all within the normal range, which is very important for judging the clinical value of this study. Since the red blood cells used were normal, they also have higher guidance for clinical pressure and temperature‐controlled transfusion. Another advantage of this study is that the red blood cells used for pressure and temperature‐controlled transfusion came from different red blood cell units, avoiding the contingency of the experimental results and greatly improving the reliability of the results. That is, pressure and temperature‐controlled transfusion at a pressure of 50 kPa and a temperature of 46°C does not cause significant red blood cell hemolysis. In clinical practice, when blood is urgently needed, it is not appropriate to wait more than 10 min before transfusion. Compared with other studies, this study directly performs pressure and temperature‐controlled transfusion after letting the 4°C refrigerated suspended red blood cells sit for 10 min at room temperature (22–23°C) and mixing well. Therefore, the results provided by this study are closer to clinical reality and have more guidance for clinical pressure and temperature‐controlled transfusion.

Although our results are meaningful, it is more important to discuss the limitations of our study. One limitation of this study is that we only tested one type of blood warming method, namely the water bath heating method. Other methods of heating red blood cells include dry heat method, infrared heating method, microwave heating method, etc. If other blood warming methods are used in the experiment, the outlet temperature at the output end may be different, which requires further experimental research. Another limitation of this study is that it only tested the maximum pressure (50 kPa) that conventional pressurisation materials can provide, whereas other studies have tested higher and lower pressures. The limitation of this study also lies in the fact that only instantaneous testing was conducted on the blood after pressure and temperature treatment; the hemolysis of the blood after being left for longer periods, such as 24 or 48 h, was not tested. At the same time, since this study is an in vitro simulation transfusion study, it is also impossible to detect whether the function of red blood cells will be affected after pressure and temperature‐controlled treatment. All these need further experimental research to verify.

Future studies on pressure and temperature‐controlled transfusion of suspended red blood cells can further test whether there are changes in red blood cell‐related indicators after 24 and 48 h of pressure and temperature‐controlled transfusion. At the same time, by detecting the function of red blood cells, the impact of pressure and temperature‐controlled transfusion on the quality of red blood cells can be further evaluated.

## AUTHOR CONTRIBUTIONS

Chunyu Feng was responsible for conducting the experiments and authored the primary manuscript text. Rui Fan was in charge of data collection, statistics, and analysis. Haimei Ma and Huan Zhang assisted in conceptualization and final manuscript review. All authors reviewed and approved the final version of the manuscript.

## FUNDING INFORMATION

This work was supported by Tsinghua Precision Medicine Foundation. Code: 10001020110.

## CONFLICT OF INTEREST STATEMENT

The authors have no competing interests.

## Data Availability

The data that support the findings of this study are available from the corresponding author upon reasonable request.
